# Montelukast Prevents Mice Against Acetaminophen-Induced Liver Injury

**DOI:** 10.3389/fphar.2019.01070

**Published:** 2019-09-18

**Authors:** Shiyun Pu, Qinhui Liu, Yanping Li, Rui Li, Tong Wu, Zijing Zhang, Cuiyuan Huang, Xuping Yang, Jinhan He

**Affiliations:** ^1^Department of Pharmacy, West China Hospital of Sichuan University and Collaborative Innovation Center of Biotherapy, Chengdu, China; ^2^Laboratory of Clinical Pharmacy and Adverse Drug Reaction, West China Hospital of Sichuan University and Collaborative Innovation Center of Biotherapy, Chengdu, China; ^3^Molecular Medicine Research Center, State Key Laboratory of Biotherapy and Cancer Center, West China Hospital of Sichuan University and Collaborative Innovation Center of Biotherapy, Chengdu, China

**Keywords:** montelukast, acetaminophen, cysteinyl leukotriene receptor 1, glutathione, JNK

## Abstract

Acetaminophen (APAP) is a widely used over-the-counter antipyretic and analgesic drug. Overdose of APAP is the leading cause of hospital admission for acute liver failure. Montelukast is an antagonist of cysteinyl leukotriene receptor 1 (Cysltr1), which protects from inflammation and oxidative stress. However, the function of montelukast in APAP-induced hepatotoxicity remains unknown. In this study, we examined whether pharmacological inhibition of Cystlr1 could protect mice against APAP-induced hepatic damage. We found that APAP treatment upregulated messenger RNA and protein levels of Cysltr1 both *in vitro* and *in vivo*. Pharmacological inhibition of Cysltr1 by montelukast ameliorated APAP-induced acute liver failure. The hepatoprotective effect of montelukast was associated with upregulation of hepatic glutathione/glutathione disulfide level, reduction in c-Jun-NH2-terminal kinase activation and oxidative stress. In mouse primary hepatocytes, inhibition of Cysltr1 by montelukast ameliorated the expression of inflammatory-related genes and APAP-induced cytotoxicity. We conclude that montelukast may be used to treat APAP-induced acute hepatic injury.

## Introduction

Acetaminophen (APAP) is a widely used over-the-counter antipyretic and analgesic drug (Lee, 2017). Although it was considered as a safe drug, APAP overdosage can lead to hepatocellular necrosis and acute liver injury (Nakagawa et al., 2008; Lee, 2017).

The majority of APAP is metabolized by conjugating enzymes UDP-glucuronosyltransferase (UGT) and sulfotransferase (SULT) in the liver to nontoxic compounds, followed by renal and biliary excretion (Yan et al., 2018). Less than 10% of APAP is bioactivated by phase I cytochrome P450 (CYP) enzymes such as CYP1A2, CYP2E1, and CYP3A4 into reactive intermediate metabolite N-acetyl-p-benzoquinone-imine (NAPQI), which causes acute liver injury (Thummel et al., 1993; Shayiq et al., 1999). NAPQI is detoxified by glutathione (GSH) to form a hazard-free metabolite (Basavarajaiah et al., 2004). With APAP overdose, GSH is depleted, and as a result, NAPQI accumulates and binds to proteins and cause oxidative stress and the activation of c-Jun-NH_2_-terminal kinase (JNK) in the liver (Nakagawa et al., 2008). JNK activation could trigger inflammation and recruitment of monocytes and neutrophils (Woolbright and Jaeschke, 2017).

The 5-lipoxygenase (5-LO) pathway plays a significant role in the pathophysiology of APAP-induced liver inflammation and injury (Hohmann et al., 2013; Pu et al., 2016). Overdose of APAP was thought to upregulate 5-LO (Suciu et al., 2016), resulting in increasing cysteinyl leukotrienes (CysLTs) secretion (Hohmann et al., 2013). Cysteinyl leukotriene receptor 1 (Cysltr1), a receptor for CysLTs, mediated various disease in humans as well as animal models. Previous reports indicated that the expression of Cysltr1 is upregulated in adenoid hypertrophy (Gao et al., 2018). Activation of Cysltr1 stimulates JNK phosphorylation (Lei et al., 2019), leading to the trigger of inflammation (Kondeti et al., 2016). Knockout of Cysltr1 prevents mice from irritant-induced asthma (McGovern et al., 2016) and also reduces colitis-associated colon cancer (Osman et al., 2017).

Montelukast, as a selective inhibitor of Cysltr1, is clinically used for the prevention and long-term treatment of asthma (Lynch et al., 1999). Several studies investigated that montelukast has an antioxidant effect in intestinal ischemia–reperfusion injury (Wu et al., 2015) and also reduces cardiac damage (Khodir et al., 2016). The beneficial effects of montelukast have also been reported in various experimental models of liver injury (El-Boghdady et al., 2017; Hegab et al., 2018). However, the mechanism of montelukast in APAP-induced hepatotoxicity remains unknown. Under the light of this information, we investigated whether the pharmacological inhibition of Cysltr1 by montelukast in mice could protect against APAP-induced hepatotoxicity.

## Materials and Methods

### Chemicals and Reagents

APAP and dimethylsulfoxide (DMSO) was purchased from Sigma-Aldrich (St. Louis, USA). Montelukast and zafirlukast were purchased from meilunbio (Dalian, China). GSH/glutathionedisulfide(GSSG)assaykit was obtained from the Nanjing Jiancheng Bioengineering Institute (Nanjing, China). The lactate dehydrogenase (LDH) cytotoxicity assay kit and the mitochondrial membrane potential assay kit with JC-1 were offered by the Beyotime Institute of Biotechnology (Shanghai, China). Primary antibodies according Cysltr1 and β-actin were purchased from Abclonal (Wuhan, China), and the secondary antibody was purchased from Jackson ImmunoResearch (PA, USA).

### Animals

This study used 8-week-old C57BL/6J mice (22–25 g), which were randomly selected for this experimental study. The acute hepatic injury was induced by oral administration of APAP (200 mg/kg) before 16 h fasting as described (Saini et al., 2011; Pu et al., 2016). For therapeutic experiment, a dose of 3 mg/kg (Hamamoto et al., 2017) of montelukast was prepared in a 0.5% carboxy methyl cellulose. Mice were gavaged in a volume of 100 μl at 1 h after APAP administration. Mice were killed by CO_2_ at 12 h after APAP administration, and blood and liver tissue were harvested for histology. All mice in experiment were housed at West China Hospital, Sichuan University in accordance with the guidelines of the animal care utilization committee of the institute. Food and water were made freely available to the mice, except where otherwise state.

### Serum Isolation and Alanine Transaminase and Aspartate Aminotransferase Detection

Blood was placed at 4°C for 1 h, then centrifuged at 3,000×*g* for 10 min. The serum was collected from the supernatant and was separately preserved at −80°C. Serum alanine transaminase (ALT) and aspartate aminotransferase (AST) levels were detected using commercial assay kits (BioSino Bio-Technology & Science Inc.).

### H&E Staining

The smallest lobe of the livers was removed and immediately fixed in 10% neutral-buffered formalin and embedded in paraffin, sectioned at 4 μm. For H&E staining, liver sections were stained with hematoxylin and eosin. Samples were visualized under a light microscope (Nikon, Japan).

### Hepatic GSH/GSSG Detection

Mice were killed at different time points after administration of APAP. Livers were isolated and immediately removed surface blood in saline, then homogenized in 5% trichloroacetic acid, then centrifuged at 3,500 rpm for 10 min. The supernatant was used to detect liver GSH/GSSG level by hepatic GSH/GSSG assay kit (Nanjing Jiancheng Bioengineering Institute, China).

### Detection of Liver H_2_O_2_ Level and Thiobarbituric Acid Reactive Substances Production

H_2_O_2_ level and thiobarbituric acid reactive substances (TBARS) in liver were measured as described (Pu et al., 2016).

### Isolation and Treatment of Primary Mouse Hepatocytes

Hepatocytes were isolated from 6-week-old C57 BL/6J mice and cultured as described (Kizu et al., 2015; Furuta et al., 2016). Montelukast was dissolved in DMSO, and DMSO was used a control. APAP was dissolved in high-glucose Dulbecco’s modified Eagle’s medium, which was supplemented with 2% fetal bovine serum. For therapeutic experiment, primary hepatocytes were pretreated with montelukast (1, 5, and 10 μM) or vehicle (0.02% DMSO) 1 h before APAP (2.5 mM) administration (Furuta et al., 2016).

### Cell Death

Cell death was measured using the LDH cytotoxicity assay kit (Beyotime, China) and the mitochondrial membrane potential assay kit (Beyotime, China) according to the manufacturers’ recommendations. For the LDH release detection, Triton X-100, 1% (*w*/*v*), was used as a positive control. The percentage of LDH released was calculated as a ratio of the positive control LDH released (Furuta et al., 2016). For the mitochondrial membrane potential detection, the cells were incubated with 5 mg/L JC-1 dye for 30 min at 37°C in the dark and washed twice with the dye buffer. Then, the cells were quickly subjected to a fluorescence microscope for captured red or green fluorescence (Chen et al., 2018).

### RT-PCR Analysis

Total liver RNA and cellular RNA were extracted using the Trizol Reagent (Invitrogen, Carlsbad, CA), and 1,000 ng of RNA was reverse transcribed to complementary DNA using a PrimeScript RT reagent Kit (Takara RR037A). Quantitative, real-time PCR was performed on the CFX96 real-time system (Biorad) using either SYBR Green Mix (Biorad). All of the primers used with SYBR green were designed to span at least one exon to minimize the possibility of nonspecific amplification from the genomic DNA. The expression of *18s* gene was used as a housekeeping gene to normalize data. Specific primer sequences are in **Supplementary Table 1**. Relative messenger RNA (mRNA) expression was quantified using the comparative CT (Ct) method and expressed as 2^ (−∆∆Ct). Amplification specificity was evaluated by determining the product melting curve. Results are expressed as indicated in the figure legends. The following program was used: one step at 95°C for 2 min, 40 cycles of denaturation at 95°C for 30 s, and annealing and elongation at 60°C for 45 s.

### Western Blotting

Western blotting analyses were performed with protein extracts from liver homogenates (50 μg) using anti-p-ERK (1:2,000 dilution, Santa Cruz), anti-ERK (1:2,000 dilution, Santa Cruz), anti-p-JNK (1:1,000 dilution, CST), and anti-JNK (1:1,000 dilution, CST) antibodies. Immunoreactive bands were visualized on nitrocellulose membranes using alkaline phosphatase-conjugated antimouse or rabbit antibody and the Odyssey detection system (LI-COR, USA).

### Statistical Analysis

Experiments were repeated at least three times with similar results. Quantitative results are expressed as the mean ± SEM. Statistical significance was determined by Student’s unpaired two-tailed *t* test or one-way ANOVA multiple comparison test. *P* < 0.05 was considered statistically significant.

## Results

### APAP Induced Cysltr1 Expression Both *In Vivo* and *In Vitro*

**Figure 1:**
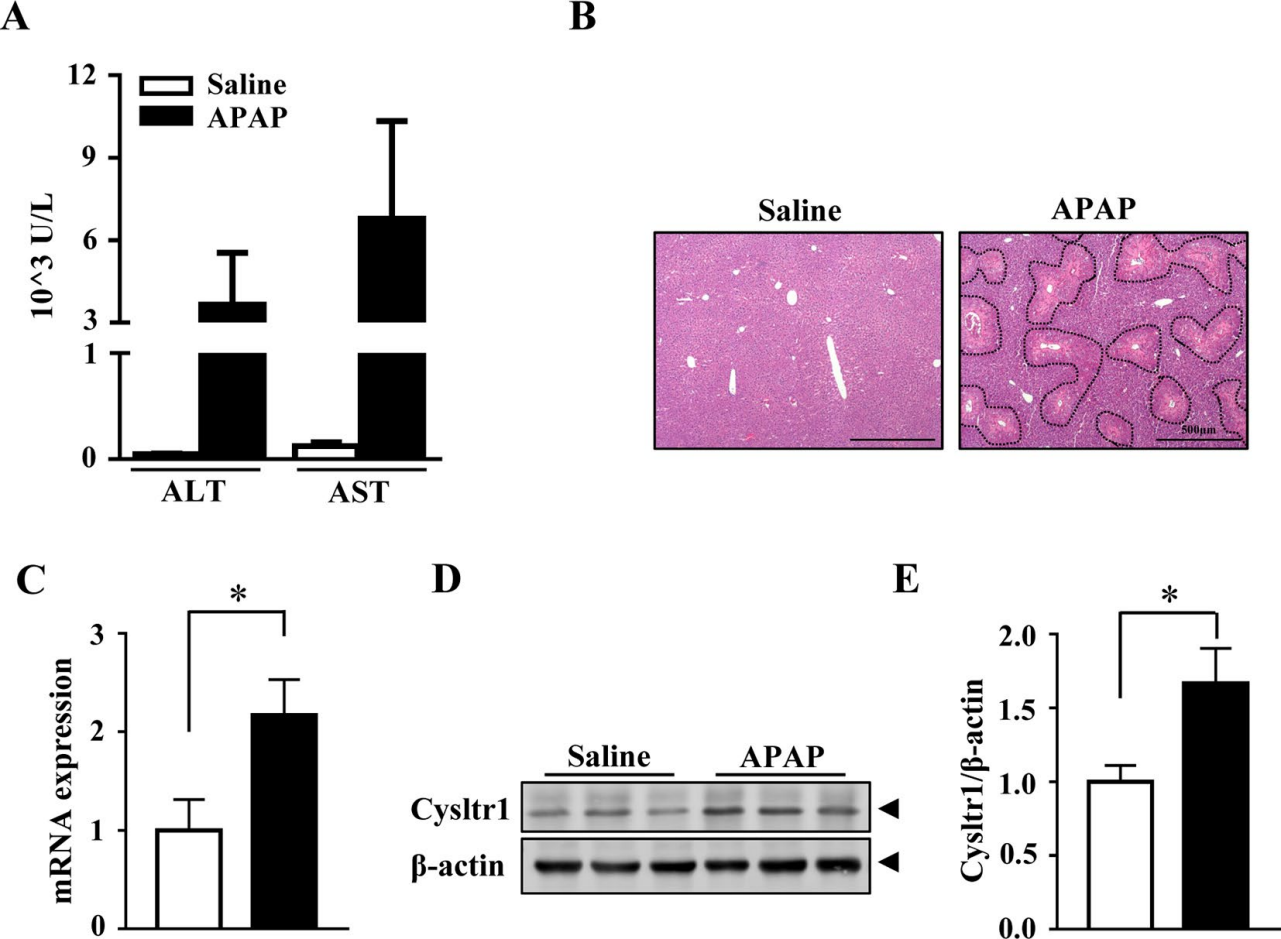
Acute acetaminophen (APAP) treatment upregulated *Cysltr1* expression *in vivo*. C57BL/6J mice fasted overnight, then were orally administered with saline or APAP (200 mg/kg) for 12 h. Data are mean ± SEM, *n* = 5 for saline group, *n* = 6 for APAP group. **(A)** Detection of serum alanine transaminase (ALT) and aspartate aminotransferase (AST). **(B)** H&E staining for livers from saline or APAP-treated mice. APAP-induced centrilobular necrosis was indicated by dotted line. **(C)** Real-time PCR analysis of hepatic messenger RNA (mRNA) expression of *Cysltr1*. **(D)** Western blot analysis of Cysltr1. **(E)** Quantification of Cysltr1 to β-actin. Data are mean ± SEM, *n* = 5, *p < 0.05.

To examined whether APAP affected the expression of *Cysltr1*, C57 BL/6J mice underwent gavage with saline or APAP (200 mg/kg) after 16 h fasting. As expected, compared to saline treatment, acute APAP treatment induced liver damage (**Figures 1A, B**). The mRNA and protein levels of *Cysltr1* were significantly upregulated in APAP-treated mice liver compared with vehicle group (**Figures 1C–E**). In contrast, *Blt1* (LTB_4_ receptor 1) was slightly decreased after APAP treatment (**Supplementary Figure 1**). APAP did not affect the expression of other leukotriene receptors such as *Blt2* and *Cysltr2* (**Supplementary Figure 1**).

We then isolated primary hepatocytes from C57/BL6J mice and assessed the mRNA and protein levels of *Cysltr1* after APAP administration *in vitro*. Similarly, the mRNA and protein level of *Cysltr1* were increased in APAP-treated hepatocytes compared with the vehicle group (**Supplementary Figures 2A, C, D**). However, the expression of *Blt1*, *Blt2*, and *Cysltr2* did not change after APAP administration (**Supplementary Figures 2A, B**).

### Pharmacological Inhibition of Cysltr1 Prevented Acetaminophen-Induced Liver Injury

**Figure 2:**
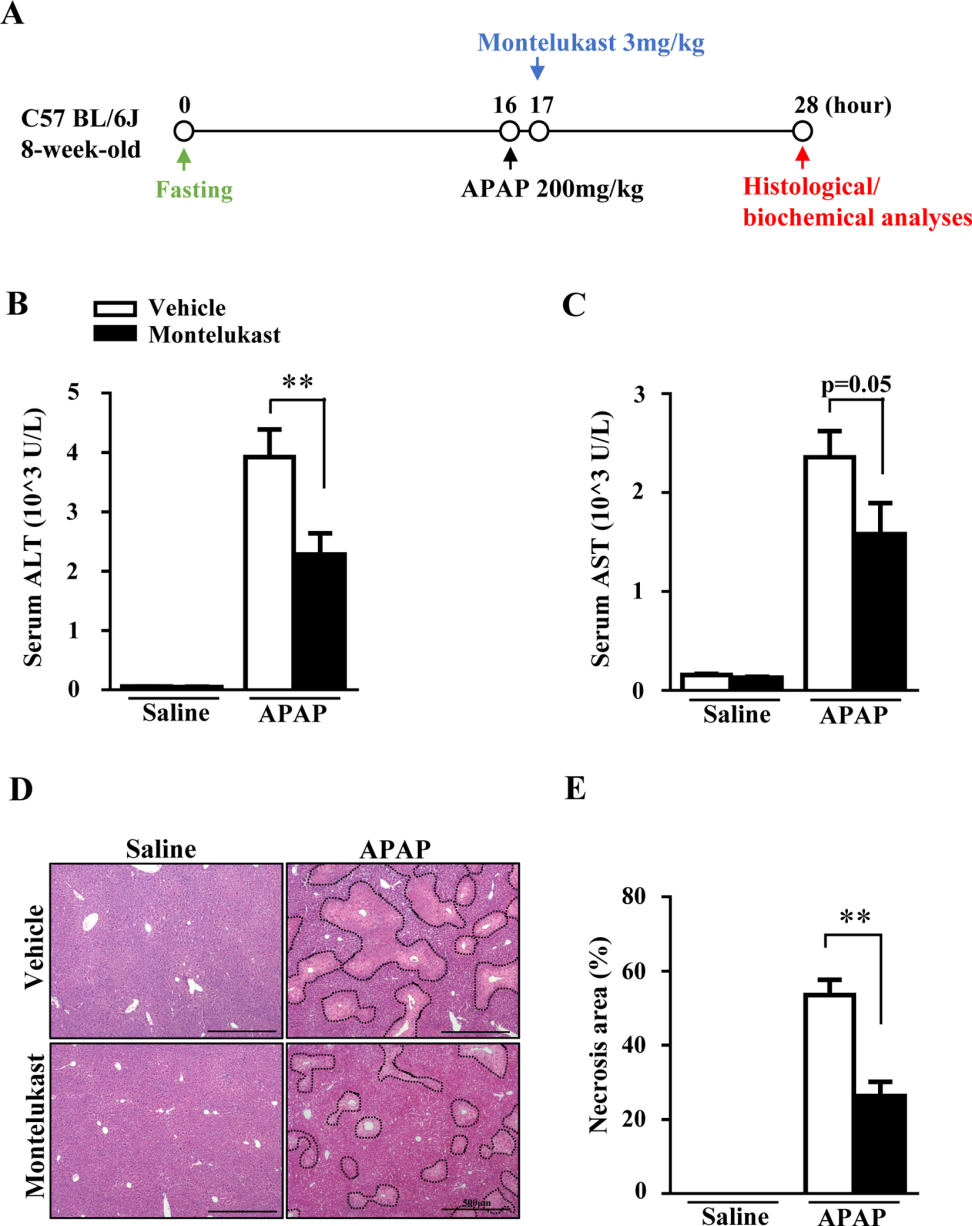
Pharmacological inhibition of Cysltr1 protected against APAP-induced hepatotoxicity. **(A)**. Schedule of montelukast administration in APAP-overdose mice. Montelukast (3 mg/kg) or vehicle were administered 1 h after APAP treatment. At 12 h after APAP administration, mice were killed, and blood and liver tissues were collected. Serum levels of ALT **(B)** and AST **(C)**. **(D)** H&E staining of liver sections from APAP- or saline-treated mice. APAP-induced centrilobular necrosis was indicated by dotted line. **(E)** Quantification of liver necrosis area. Data are mean ± SEM, *n* = 5 for saline groups, *n* = 7 for APAP groups, **p < 0.01.

The increased expression of *Cysltr1* in APAP overdose-treated mouse liver prompted us to determine whether pharmacological inhibition of Cysltr1 would affect APAP-induced liver toxicity. C57BL/6J mice were treated with vehicle or the Cystlr1 antagonist, montelukast (3 mg/kg), 1 h after saline or APAP administration (**Figure 2A**). Mice were killed 12 h after saline or APAP treatment, and blood and liver tissues were harvested. Montelukast treatment significantly decreased serum levels of ALT and AST (**Figures 2B, C**) and alleviated liver damage as indicated by H&E staining in APAP-treated groups (**Figures 2D, E**). Montelukast has no effect on saline-treated groups (**Figures 2B–E**). These results suggested that inhibition of Cysltr1 protected mice from APAP-induced liver injury.

### Montelukast Treatment Did Not Alter APAP Phase I/II Enzymes and Hepatic Transporters

Resistance to APAP toxicity in montelukast-treated mice led us to speculate that pharmacological inhibition of Cysltr1 may alter the formation of toxicity-induced metabolites and/or promote APAP clearance. To investigate whether montelukast administration affected APAP metabolism, we first measured phase I enzymes, which are responsible for the formation of toxic APAP metabolites. However, the expression of *Cyp1a2*, *Cyp2e1*, and *Cyp3a11* in livers were nearly comparable between montelukast-treated and vehicle group (**Supplementary Figures 3A–C**). Phase II enzymes were known to detoxify APAP. However, the expression of UDP-glucuronosyltransferase 1A1 (*Ugt1a1*) and sulfotransferase 2A1 (*Sult2a1*) did not change in montelukast-treated mice (**Supplementary Figures 3D, E**). Transporters were also associated with APAP-induced acute liver damage. However, montelukast administration did not alter the expression of transporters (**Supplementary Figures 4A–C**).

### Montelukast Treatment Upregulated Hepatic GSH Level and Gstα2 Expression.

**Figure 3:**
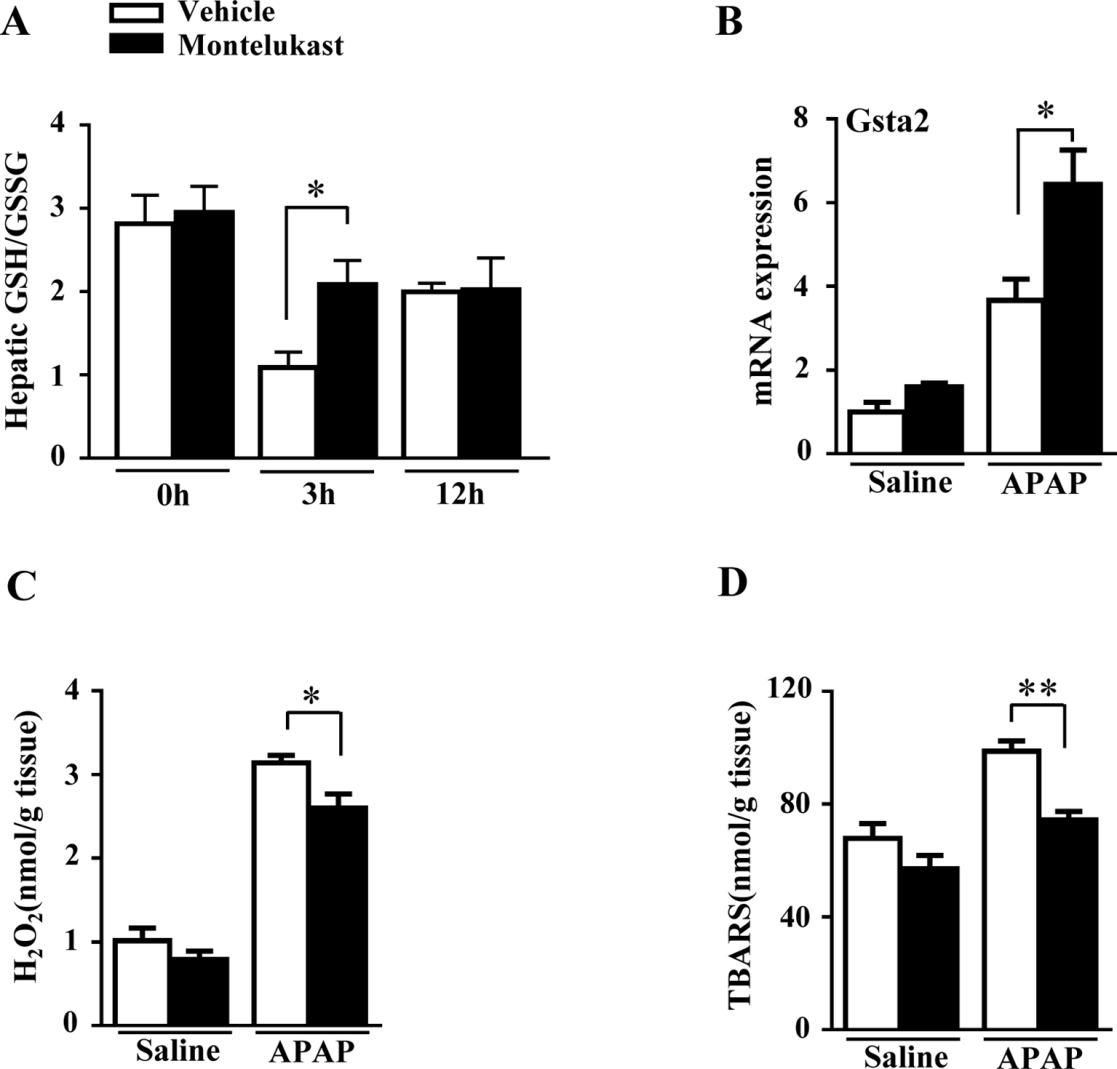
Montelukast treatment maintained hepatic GSH level and reduced reactive oxygen species production in APAP treated mice. **(A)** Detection of hepatic glutathione (GSH)/glutathione disulfide (GSSG) level. **(B)** Real-time PCR analysis of hepatic mRNA expression of GSTa2. **(C)** Hepatic H_2_O_2_ level. **(D)** APAP-induced thiobarbituric acid reactive substances (TBARS) level. Data are mean ± SEM, *n* = 5 for saline groups, *n* = 7 for APAP groups, *p < 0.05 , **p < 0.01.

GSH plays a critical role in detoxifying the reactive intermediate of APAP (Nakagawa et al., 2008). With overdoses of APAP administration, hepatic GSH/GSSG level was markedly depleted in the vehicle group at 3 h and recovered at 12 h (**Figure 3A**). However, hepatic GSH/GSSG level was higher in montelukast-treated mice at 3 h (**Figure 3A**). There was no difference in hepatic GSH/GSSG levels between vehicle and montelukast-treated groups at 12 h after APAP administration. Glutathionylation is catalyzed by a group of enzymes called the glutathione S-transferase (GST). We found enhanced *Gsta2* expression in montelukast treated liver (**Figure 3B**). However, the mRNA levels of *GSTa1*, *GSTm1*, and *GSTm2* were unaffected (**Supplementary Figures 4D–F**).

### Montelukast Decreased ROS Levels in Mice

Hepatic GSH depletion accumulates APAP metabolites covalently bound to protein, which cause oxidative stress and lipid peroxidation (Nakagawa et al., 2008). We evaluated the H_2_O_2_ and TBARS level in mice treated with saline or APAP. We found that APAP greatly increased hepatic H_2_O_2_ and TBARS level in both vehicle and montelukast-treated mice (**Figures 3C, D**). However, montelukast decreased both hepatic H_2_O_2_ and TBARS level compared with vehicle-treated mice (**Figures 3C, D**).

### Pharmacological Inhibition of Cysltr1 by Montelukast Prevented Acetaminophen-Induced Liver Inflammation and JNK Activation

**Figure 4:**
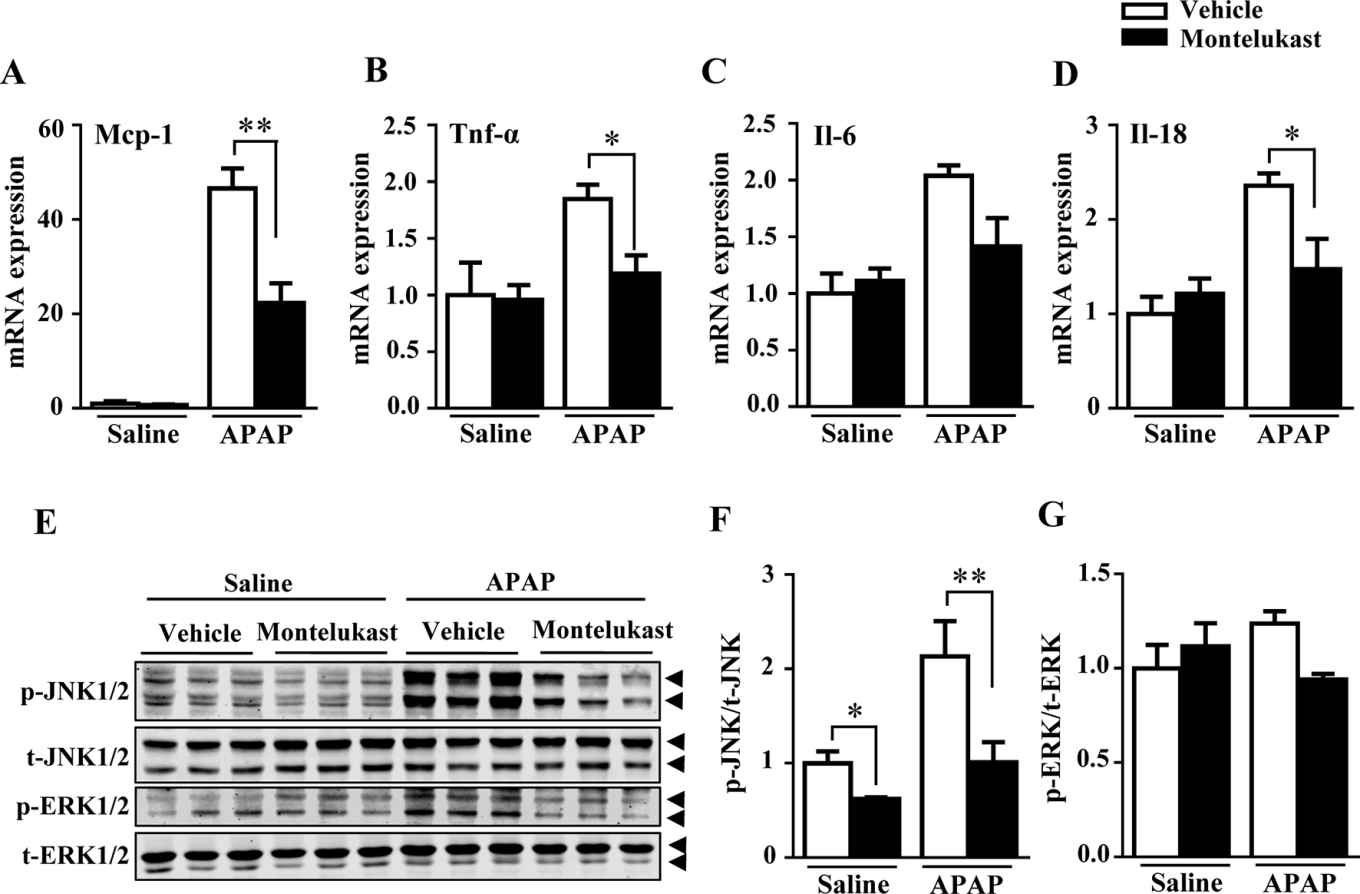
Pharmacological inhibition of cysltr1 decrease APAP-induced hepatic inflammation. **(A**–**D)** Real-time PCR analysis of hepatic inflammatory cytokine genes expression, *n* = 5 for saline groups, *n* = 7 for APAP groups. **(E)** Western blotting analysis ERK and JNK phosphorylation. **(F** and **G)** Quantification of phosphorylated JNK to total JNK and phosphorylated ERK to total ERK. Data are mean ± SEM, *n* = 3 for each groups, *p < 0.05, **p < 0.01. Experiments were repeated three times with similar results.

With overdoses of APAP, hepatic GSH depletion resulted in APAP metabolites covalently bound to protein, which further exacerbates hepatic toxicity by causing inflammatory responses (Luster et al., 2000). In our study, levels of the inflammatory cytokines, including *Mcp-1*, *TNF-α*, *Il-6*, and *Il-18*, were increased by APAP administration as expected (**Figures 4A–D**). Montelukast administration alleviated APAP-induced inflammation (**Figures 4A–D**). The phosphorylation of JNK1/2 and ERK1/2, mediators of inflammation, were increased by APAP overdosage (Arthur and Ley, 2013). Montelukast suppressed the phosphorylation levels of JNK1/2 after APAP administration (**Figures 4E, F**). Meanwhile, pharmacological inhibition of Cysltr1 slightly inhibited ERK1/2 activation in APAP-treated groups (**Figures 4E, G**).

### Pharmacological Inhibition of Cysltr1 Protects Mouse Primary Hepatocytes From APAP-Induced Cell Death.

**Figure 5:**
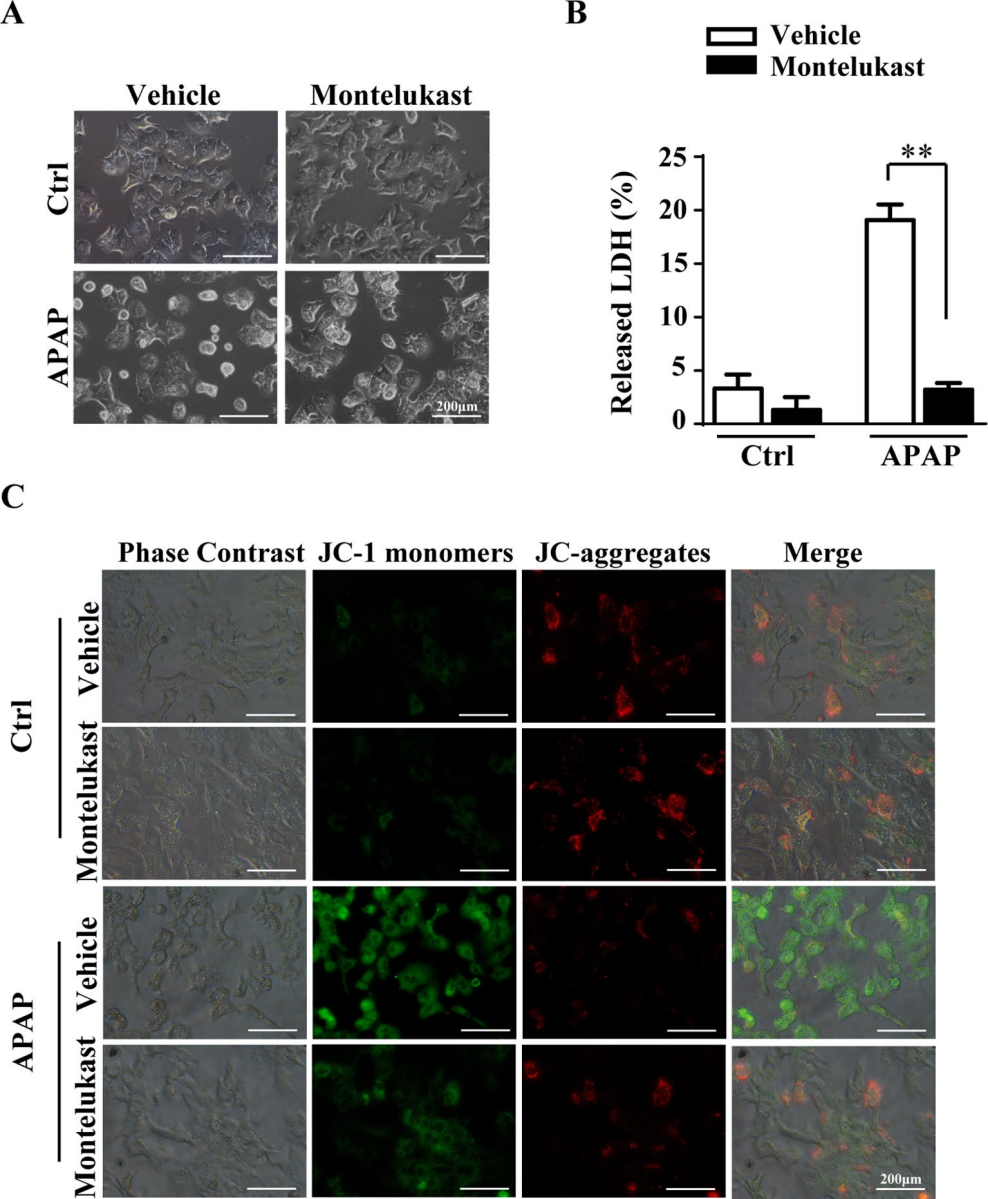
Montelukast inhibit APAP-induced cell damage. Primary hepatocytes were pretreated with montelukast (5 μM) or vehicle (DMSO) 1 h before APAP (2.5 mM) administration. **(A)** Representative morphological images of primary hepatocytes treated with APAP for 24 h. **(B)** Quantification of LDH released into the culture medium of primary hepatocyte after treatment with 2.5 mM of APAP for 24 h. Data are mean ± SEM, *n* = 3 for each group, **p < 0.01. **(C)** Primary hepatocytes were incubated with 5 mg/l JC-1 dye for 30 min at 37°C in the dark and washed twice with the dye buffer. Then, the cells were quickly subjected to a fluorescence microscope for captured red or green fluorescence. Experiments were repeated three times with similar results.

To further confirm the protective role of montelukast in APAP-induced hepatotoxicity, primary hepatocytes were isolated and pretreated with montelukast 1 h before APAP administration. APAP treatment remarkably increased cytotoxicity as expected (**Figures 5A, B**). Montelukast decreased APAP-induced LDH released at concentrations of 5 and 10 μM (**Supplementary Figure 5A**). It seems that montelukast has little effect on the expression of Cysltr1 (**Supplementary Figure 5B**). However, montelukast dramatically reversed APAP-induced mitochondrial membrane potential decreasing (**Figure 5C**). Zafirlukast, another kind of Cysltr1 antagonist, also improved APAP-induced hepatocyte death (**Supplementary Figures 6A, B**). Moreover, montelukast inhibited LTD_4_, a Cystlr1 agonist, induced hepatocytes damage (**Supplementary Figure 7**), and thus partly improved APAP-induced hepatotoxicity.

## Discussion

In this study, we showed that inhibition of Cysltr1 protected mice against APAP-induced liver damage. Montelukast postadministration inhibited APAP-induced hepatic oxidative stress and necrosis. Mechanistically, inhibition of Cysltr1 in APAP-treated mice induced the expression of *GSTa2*, which promotes NAPQI detoxification. Compared to the vehicle group, these protective effects were also associated with higher hepatic GSH/GSSG ratio and lower oxidative stress and JNK activity in montelukast-treated group. Montelukast administration also inhibited the inflammatory response with APAP overdose.

Phase II enzymes eliminate most APAP and protect livers from toxicity. The majority of APAP is metabolized by UGT and SULT, then excreted in the urine (McGill and Jaeschke, 2013). Upregulated of UGT and SULT would be helpful to prevent toxicity of APAP overdosage (McGill and Jaeschke, 2013). However, montelukast did not alter the expression of *Ugt1a1* and *Sult2a1* in our study. Phase I metabolic enzymes and transporters also remained unchanged.

Our results showed that protective effect of montelukast in APAP-induced liver damage was associated with an increased level of hepatic GSH, which was thought to accumulate phase I metabolites detoxification. Glutathionylation is catalyzed by a group of enzymes called the GST. Gstm-null mice are found to be resistant to APAP (Arakawa et al., 2012). Upregulation of Gsta is also thought to prevent APAP-induced liver failure (Coles and Kadlubar, 2005; Gum and Cho, 2013). We also showed that montelukast increased *GSTa2* expression after APAP administration, which may improve APAP-induced hepatotoxicity.

Recent studies have shown that montelukast has effects of antioxidant and anti-inflammation in various experimental models (Dengiz et al., 2007; Vollmar and Menger, 2011; Khodir et al., 2016). Pharmacological inhibition of Cysltr1 protected aluminum-overload-induced liver injury through PI3K/AKT/mTOR pathway (Hu et al., 2018). At overdoses of APAP administration, hepatic GSH was depleted and APAP metabolites covalently bound to protein, which further exacerbates hepatic toxicity by triggering oxidative stress and JNK activation (Luster et al., 2000). Inhibition of Cysltr1 by montelukast treatment prevented APAP-induced oxidative stress and JNK activation. Activation of JNK mediates hepatocyte necrosis and trigger inflammation in APAP-induced liver injury model (Jaeschke et al., 2012; Woolbright and Jaeschke, 2017). Several studies suggested that inflammation plays a role in hepatocyte necrosis and is thought to promote liver injury (Hoque et al., 2012). The decreased activity of JNK and inflammation may contribute to protective effect of montelukast in APAP overdose model.

Studies have reported upregulation of 5-LO pathway in APAP-induced liver injury (Suciu et al., 2016). Activation of the 5-LO pathway leads to the formation of leukotrienes from arachidonic acid (Zhao et al., 2004). Leukotrienes are thought to be involved in the production of inflammation and lead to liver injury (Samuelsson et al., 1987; Kaminski, 1989; Martinez-Clemente et al., 2010). We also found LTD_4_-induced hepatocytes injury by promoting LDH released and decreasing mitochondrial membrane potential (**Supplementary Figure 7**). Knockout or inhibition of 5-LO could prevent APAP-induced liver inflammation and liver injury (Martinez-Clemente et al., 2010; Hohmann et al., 2013). Meanwhile, knockout or inhibition of leukotriene receptors is an effective way to treat tissue damage caused by leukotrienes (Beller et al., 2004). Several leukotriene receptors are identified. They are Blt1, Blt2, Cysltr1, Cysltr2, and Cysltr3. Functional consequences of Cysltr3 activation are not well defined (Evans et al., 2008). Although it has been reported that blocking Blt1 signaling protected APAP-induced hepatotoxicity through preventing excessive accumulation of hepatic neutrophils (Kojo et al., 2016), Blt1 receptor antagonists have no further development (Evans et al., 2008). Montelukast inhibited LTD_4_-induced hepatocytes damage (**Supplementary Figure 7**) and thus partly improved APAP-induced hepatotoxicity. Therefore, montelukast administration may be a strategy for inhibiting APAP-induced liver injury.

It was attractive that APAP promoted Cysltr1 expression both *in vivo* and *in vitro*. The expression of Cysltr1 was significantly induced in the hepatic ischemia–reperfusion injury and aluminum-overload-induced liver injury (Wu et al., 2015; Hu et al., 2018). The elevated expression of Cysltr1 has been shown to participate the pathological progress of these diseases. In our study, we also found that Cysltr1 expression was higher in model of APAP-overdose-induced liver injury than the saline group. Blocking Cysltr1 by montelukast-alleviated APAP overdose caused oxidative stress and inflammation. *In vitro*, it was reported that TNF-α, an activator of necrosis, could stimulate Cysltr1 expression in human bronchi and thus promotes hyper-responsiveness (Khaddaj-Mallat et al., 2016). We also found that APAP upregulated Cysltr1 in primary hepatocytes, and it thereby enlarged hepatotoxicity. Future study is needed to study the mechanism of Cysltr1 upregulation under APAP overdose.

N-acetylcysteine (NAC) is thought to be useful in the management of APAP-induced liver injury (Brok et al., 2006). In APAP-treated liver, NAC is deacetylated to form L-cysteine, an essential amino acid for the synthesis of GSH (Smilkstein et al., 1991). One report compared the therapeutic effects of NAC and montelukast on APAP-induced hepatotoxicity and found that montelukast had better therapeutic effect than NAC did (Icer et al., 2016). However, they did not explain the mechanism of montelukast administration in APAP-induced hepatic damage. We observed an increased level of liver GSH/GSSG level, a reduced level of oxidative stress, JNK phosphorylation, and a decreased level of inflammation. These results will elucidate the protective mechanism of montelukast on APAP-induced liver injury. In the future, the prophylactic effect of montelukast should be further evaluated in APAP-induced liver damage.

In summary, the present study demonstrated that pharmacological inhibition of Cysltr1 by montelukast conferred resistance to APAP-induced hepatotoxicity. The protective effect of montelukast was, at least in part, attributed to upregulation of hepatic GSH/GSSG, suppression of oxidative stress and JNK pathway. Targeted inhibition of the Cysltr1 by montelukast may be a potential treatment strategy in APAP-induced hepatotoxicity.

## Ethics Statement

This study was carried out in accordance with the recommendations of the guidelines of the animal care utilization committee of the institute, the Institutional Animal Care and Use Committee of Sichuan University. The protocol was approved by the Institutional Animal Care and Use Committee of Sichuan University.

## Author Contributions

SP and JH participated in research design. SP, QL, RL, ZZ, XY, and CH conducted experiments. SP, ZZ, and TW performed data analysis. SP, YL, and JH wrote or contributed to the writing of the manuscript.

## Funding

This work was supported by the National Natural Science Foundation of China (81873662, 81870599, and 81603035), China Postdoctoral Fellowship (2017M612981), Young Scientist Fellowship of Sichuan University (2017SCU11026), and Postdoctoral Fellowship of Sichuan University (2017SCU12036).

## Conflict of Interest Statement

The authors declare that the research was conducted in the absence of any commercial or financial relationships that could be construed as a potential conflict of interest.

## Abbreviation

APAP, acetaminophen; MK, montelukast; Cysltr1, Cysteinyl leukotriene receptor 1; JNK, c-Jun-NH2-terminal kinase; NAPQI, N-acetyl-*p*-benzoquinone-imine; 5-LO, 5-lipoxygenase.

## References

[B1] Arakawa S. Maejima T. Fujimoto K. Yamaguchi T. Yagi M. Sugiura T. (2012). Resistance to acetaminophen-induced hepatotoxicity in glutathione S-transferase Mu 1-null mice. J. Toxicol. Sci. 37 (3), 595–605. 10.2131/jts.37.595 22687999

[B2] Arthur J. S. Ley S. C. (2013). Mitogen-activated protein kinases in innate immunity. Nat. Rev. Immunol. 13 (9), 679–692. 10.1038/nri3495 23954936

[B3] Basavarajaiah S. Sigston P. Budack K. (2004). Severe salicylate poisoning treated conservatively. J. R. Soc. Med. 97 (12), 587–588. 10.1258/jrsm.97.12.587 15574860 PMC1079675

[B4] Beller T. C. Friend D. S. Maekawa A. Lam B. K. Austen K. F. Kanaoka Y. (2004). Cysteinyl leukotriene 1 receptor controls the severity of chronic pulmonary inflammation and fibrosis. Proc. Natl. Acad. Sci. U. S. A. 101 (9), 3047–3052. 10.1073/pnas.0400235101 14970333 PMC365742

[B5] Brok J. Buckley N. Gluud C. (2006). Interventions for paracetamol (acetaminophen) overdose. Cochrane Database Syst. Rev. 2,CD003328. 10.1002/14651858.CD003328.pub2 16625578

[B6] Chen L. Li W. Qi D. Lu L. Zhang Z. Wang D. (2018). Honokiol protects pulmonary microvascular endothelial barrier against lipopolysaccharide-induced ARDS partially via the Sirt3/AMPK signaling axis. Life Sci. 210, 86–95. 10.1016/j.lfs.2018.08.064 30171880

[B7] Coles B. F. Kadlubar F. F. (2005). Human alpha class glutathione S-transferases: genetic polymorphism, expression, and susceptibility to disease. Methods Enzymol. 401, 9–42. 10.1016/S0076-6879(05)01002-5 16399377

[B8] Dengiz G. O. Odabasoglu F. Halici Z. Suleyman H. Cadirci E. Bayir Y. (2007). Gastroprotective and antioxidant effects of amiodarone on indomethacin-induced gastric ulcers in rats. Arch. Pharm. Res. 30 (11), 1426–1434. 10.1007/BF02977367 18087811

[B9] El-Boghdady N. A. Abdeltawab N. F. Nooh M. M. (2017). Resveratrol and montelukast alleviate paraquat-induced hepatic injury in mice: modulation of oxidative stress, inflammation, and apoptosis. Oxid. Med. Cell Longev. 2017, 9396425. 10.1155/2017/9396425 29201275 PMC5671749

[B10] Evans J. F. Ferguson A. D. Mosley R. T. Hutchinson J. H. (2008). What’s all the FLAP about?: 5-lipoxygenase-activating protein inhibitors for inflammatory diseases. Trends Pharmacol. Sci. 29 (2), 72–78. 10.1016/j.tips.2007.11.006 18187210

[B11] Furuta K. Yoshida Y. Ogura S. Kurahashi T. Kizu T. Maeda S. (2016). Gab1 adaptor protein acts as a gatekeeper to balance hepatocyte death and proliferation during acetaminophen-induced liver injury in mice. Hepatology 63 (4), 1340–1355. 10.1002/hep.28410 26680679

[B12] Gao W. Li J. Li Q. An S. (2018). CYSLTR1 promotes adenoid hypertrophy by activating ERK1/2. Exp. Ther. Med. 16 (2), 966–970. 10.3892/etm.2018.6282 30116346 PMC6090203

[B13] Gum S. I. Cho M. K. (2013). The amelioration of N-acetyl-p-benzoquinone imine toxicity by ginsenoside Rg3: the role of Nrf2-mediated detoxification and Mrp1/Mrp3 transports. Oxid. Med. Cell Longev. 2013, 957947. 10.1155/2013/957947 23766864 PMC3666202

[B14] Hamamoto Y. Ano S. Allard B. O’Sullivan M. McGovern T. K. Martin J. G. (2017). Montelukast reduces inhaled chlorine triggered airway hyperresponsiveness and airway inflammation in the mouse. Br. J. Pharmacol. 174 (19), 3346–3358. 10.1111/bph.13953 28718891 PMC5595758

[B15] Hegab I. I. El-Horany H. E. Elbatsh M. M. Helal D. S. (2018). Montelukast abrogates prednisolone-induced hepatic injury in rats: modulation of mitochondrial dysfunction, oxidative/nitrosative stress, and apoptosis. J. Biochem. Mol. Toxicol. 33, e22231. 10.1002/jbt.22231 30276927

[B16] Hohmann M. S. Cardoso R. D. Pinho-Ribeiro F. A. Crespigio J. Cunha T. M. Alves-Filho J. C. (2013). 5-Lipoxygenase deficiency reduces acetaminophen-induced hepatotoxicity and lethality. Biomed. Res. Int. 2013, 627046. 10.1155/2013/627046 24288682 PMC3832964

[B17] Hoque R. Sohail M. A. Salhanick S. Malik A. F. Ghani A. Robson S. C. (2012). P2X7 receptor-mediated purinergic signaling promotes liver injury in acetaminophen hepatotoxicity in mice. Am. J. Physiol. Gastrointest Liver Physiol. 302 (10), G1171–G1179. 10.1152/ajpgi.00352.2011 22383490 PMC3362096

[B18] Hu C. Yang J. He Q. Luo Y. Chen Z. Yang L. (2018). CysLTR1 blockage ameliorates liver injury caused by aluminum-overload via PI3K/AKT/mTOR-mediated autophagy activation in vivo and in vitro. Mol. Pharm. 15 (5), 1996–2006. 10.1021/acs.molpharmaceut.8b00121 29634275

[B19] Icer M. Zengin Y. Gunduz E. Dursun R. Durgun H. M. Turkcu G. (2016). Is montelukast as effective as N-acetylcysteine in hepatic injury due to acetaminophen intoxication in rats? Exp. Toxicol. Pathol. 68 (1), 55–59. 10.1016/j.etp.2015.09.008 26462568

[B20] Jaeschke H. Williams C. D. Ramachandran A. Bajt M. L. (2012). Acetaminophen hepatotoxicity and repair: the role of sterile inflammation and innate immunity. Liver Int. 32 (1), 8–20. 10.1111/j.1478-3231.2011.02501.x 21745276 PMC3586825

[B21] Kaminski D. L. (1989). Arachidonic acid metabolites in hepatobiliary physiology and disease. Gastroenterology 97 (3), 781–792. 10.1016/0016-5085(89)90655-0 2666254

[B22] Khaddaj-Mallat R. Sirois C. Sirois M. Rizcallah E. Marouan S. Morin C. (2016). Pro-resolving effects of resolvin D2 in LTD4 and TNF-alpha pre-treated human bronchi. PLoS One 11 (12), e0167058. 10.1371/journal.pone.0167058 27935998 PMC5148597

[B23] Khodir A. E. Ghoneim H. A. Rahim M. A. Suddek G. M. (2016). Montelukast attenuates lipopolysaccharide-induced cardiac injury in rats. Hum. Exp. Toxicol. 35 (4), 388–397. 10.1177/0960327115591372 26089034

[B24] Kizu T. Yoshida Y. Furuta K. Ogura S. Egawa M. Chatani N. (2015). Loss of Gab1 adaptor protein in hepatocytes aggravates experimental liver fibrosis in mice. Am. J. Physiol. Gastrointest Liver Physiol. 308 (7), G613–G624. 10.1152/ajpgi.00289.2014 25617348

[B25] Kojo K. Ito Y. Eshima K. Nishizawa N. Ohkubo H. Yokomizo T. (2016). BLT1 signalling protects the liver against acetaminophen hepatotoxicity by preventing excessive accumulation of hepatic neutrophils. Sci. Rep. 6, 29650. 10.1038/srep29650 27404729 PMC4939602

[B26] Kondeti V. Al-Azzam N. Duah E. Thodeti C. K. Boyce J. A. Paruchuri S. (2016). Leukotriene D4 and prostaglandin E2 signals synergize and potentiate vascular inflammation in a mast cell-dependent manner through cysteinyl leukotriene receptor 1 and E-prostanoid receptor 3. J. Allergy Clin. Immunol. 137 (1), 289–298. 10.1016/j.jaci.2015.06.030 26255103 PMC5839097

[B27] Lee W. M. (2017). Acetaminophen (APAP) hepatotoxicity-Isn’t it time for APAP to go away? J. Hepatol. 67 (6), 1324–1331. 10.1016/j.jhep.2017.07.005 28734939 PMC5696016

[B28] Lei C. Wu S. Wen C. Li Y. Liu N. Huang J. (2019). Zafirlukast attenuates advanced glycation end-products (AGEs)-induced degradation of articular extracellular matrix (ECM). Int. Immunopharmacol. 68, 68–73. 10.1016/j.intimp.2018.12.056 30612086

[B29] Luster M. I. Simeonova P. P. Gallucci R. M. Matheson J. M. Yucesoy B. (2000). Immunotoxicology: role of inflammation in chemical-induced hepatotoxicity. Int. J. Immunopharmacol. 22 (12), 1143–1147. 10.1016/S0192-0561(00)00073-4 11137622

[B30] Lynch K. R. O’Neill G. P. Liu Q. Im D. S. Sawyer N. Metters K. M. (1999). Characterization of the human cysteinyl leukotriene CysLT1 receptor. Nature 399 (6738), 789–793. 10.1038/21658 10391245

[B31] Martinez-Clemente M. Ferre N. Gonzalez-Periz A. Lopez-Parra M. Horrillo R. Titos E. (2010). 5-Lipoxygenase deficiency reduces hepatic inflammation and tumor necrosis factor alpha-induced hepatocyte damage in hyperlipidemia-prone ApoE-null mice. Hepatology 51 (3), 817–827. 10.1002/hep.23463 20112424

[B32] McGill M. R. Jaeschke H. (2013). Metabolism and disposition of acetaminophen: recent advances in relation to hepatotoxicity and diagnosis. Pharm. Res. 30 (9), 2174–2187. 10.1007/s11095-013-1007-6 23462933 PMC3709007

[B33] McGovern T. Goldberger M. Chen M. Allard B. Hamamoto Y. Kanaoka Y. (2016). CysLT1 receptor is protective against oxidative stress in a model of irritant-induced asthma. J. Immunol. 197 (1), 266–277. 10.4049/jimmunol.1501084 27226094

[B34] Nakagawa H. Maeda S. Hikiba Y. Ohmae T. Shibata W. Yanai A. (2008). Deletion of apoptosis signal-regulating kinase 1 attenuates acetaminophen-induced liver injury by inhibiting c-Jun N-terminal kinase activation. Gastroenterology 135 (4), 1311–1321. 10.1053/j.gastro.2008.07.006 18700144

[B35] Osman J. Savari S. Chandrashekar N. K. Bellamkonda K. Douglas D. Sjolander A. (2017). Cysteinyl leukotriene receptor 1 facilitates tumorigenesis in a mouse model of colitis-associated colon cancer. Oncotarget 8 (21), 34773–34786. 10.18632/oncotarget.16718 28410235 PMC5471010

[B36] Pu S. Ren L. Liu Q. Kuang J. Shen J. Cheng S. (2016). Loss of 5-lipoxygenase activity protects mice against paracetamol-induced liver toxicity. Br. J. Pharmacol. 173 (1), 66–76. 10.1111/bph.13336 26398229 PMC4813378

[B37] Saini S. P. Zhang B. Niu Y. Jiang M. Gao J. Zhai Y. (2011). Activation of liver X receptor increases acetaminophen clearance and prevents its toxicity in mice. Hepatology 54 (6), 2208–2217. 10.1002/hep.24646 21898498 PMC3230770

[B38] Samuelsson B. Dahlen S. E. Lindgren J. A. Rouzer C. A. Serhan C. N. (1987). Leukotrienes and lipoxins: structures, biosynthesis, and biological effects. Science 237 (4819), 1171–1176. 10.1126/science.2820055 2820055

[B39] Shayiq R. M. Roberts D. W. Rothstein K. Snawder J. E. Benson W. Ma X. (1999). Repeat exposure to incremental doses of acetaminophen provides protection against acetaminophen-induced lethality in mice: an explanation for high acetaminophen dosage in humans without hepatic injury. Hepatology 29 (2), 451–463. 10.1002/hep.510290241 9918922

[B40] Smilkstein M. J. Bronstein A. C. Linden C. Augenstein W. L. Kulig K. W. Rumack B. H. (1991). Acetaminophen overdose: a 48-hour intravenous N-acetylcysteine treatment protocol. Ann. Emerg. Med. 20 (10), 1058–1063. 10.1016/S0196-0644(05)81352-6 1928874

[B41] Suciu M. Gruia A. T. Nica D. V. Azghadi S. M. Mic A. A. Mic F. A. (2016). Data on expression of lipoxygenases-5 and -12 in the normal and acetaminophen-damaged liver. Data Brief 7, 1199–1203. 10.1016/j.dib.2016.03.079 27408922 PMC4927949

[B42] Thummel K. E. Lee C. A. Kunze K. L. Nelson S. D. Slattery J. T. (1993). Oxidation of acetaminophen to N-acetyl-p-aminobenzoquinone imine by human CYP3A4. Biochem. Pharmacol. 45 (8), 1563–1569. 10.1016/0006-2952(93)90295-8 8387297

[B43] Vollmar B. Menger M. D. (2011). Intestinal ischemia/reperfusion: microcirculatory pathology and functional consequences. Langenbecks Arch. Surg. 396 (1), 13–29. 10.1007/s00423-010-0727-x 21088974

[B44] Woolbright B. L. Jaeschke H. (2017). Role of the inflammasome in acetaminophen-induced liver injury and acute liver failure. J. Hepatol. 66 (4), 836–848. 10.1016/j.jhep.2016.11.017 27913221 PMC5362341

[B45] Wu S. Zhu X. Jin Z. Tong X. Zhu L. Hong X. (2015). The protective role of montelukast against intestinal ischemia–reperfusion injury in rats. Sci. Rep. 5, 15787. 10.1038/srep15787 26497763 PMC4620564

[B46] Yan M. Huo Y. Yin S. Hu H. (2018). Mechanisms of acetaminophen-induced liver injury and its implications for therapeutic interventions. Redox Biol. 17, 274–283. 10.1016/j.redox.2018.04.019 29753208 PMC6006912

[B47] Zhao L. Moos M. P. Grabner R. Pedrono F. Fan J. Kaiser B. (2004). The 5-lipoxygenase pathway promotes pathogenesis of hyperlipidemia-dependent aortic aneurysm. Nat. Med. 10 (9), 966–973. 10.1038/nm1099 15322539

