# Supplementary material for: Montelukast Prevents Mice Against Acetaminophen-Induced Liver Injury
Source: Front Pharmacol. 2019 Sep 18;10:1070. doi: 10.3389/fphar.2019.01070 (PMC6759817; doi:10.3389/fphar.2019.01070)

Supplementary materials

Supplementary Figure 1. The mRNA level of *Blt1, Blt2* and *Cysltr2* in mice liver after acute APAP administration.
C57BL/6J mice fasted overnight, then were orally administered with saline or APAP (200 mg/kg) for 12 hours. The hepatic mRNA expression of *Blt1*, *Blt2* and *Cysltr2* were detected by RT-qPCR analysis. Data are mean ±SEM, n = 5 for saline group, n=6 for APAP group. $*P < 0.05$.

Supplementary Figure 2. APAP treatment upregulated *Cysltr1* expression *in vivo*.
A-B, Primary hepatocytes were treated with vehicle or APAP (2.5 mM) for 24 hours. The mRNA levels of *Cysltr1*, *Cysltr2*, *Blt1* and *Blt2* in primary hepatocytes were detected by RT-qPCR analysis. Data are mean ± SEM, n = 5 for each group, $*p < 0.05$. **C**, Western blot analysis of Cysltr1. Primary hepatocytes were treated with 2.5 mM APAP for 0, 3, 8 or 24 hours. **D,** Quantification of Cysltr1 to β-actin. Data are mean ± SEM, n = 5, $*p < 0.05$. Experiments were repeated 3 times with similar results.

Supplementary Figure 3. Montelukast administration did not affect APAP phase I/II metabolizing enzymes.
A-C, The hepatic mRNA levels of *Cyp1a2*, *Cyp 2a1*, and *Cyp3a11*. **D-E**, The mRNA levels of *Ugt1a1* and *Sult2a1*. Data are mean ±SEM, n = 5 for saline groups, n = 7 for APAP groups.

Supplementary Figure 4. The hepatic mRNA levels of transporters and GSTs.
A-C, The expression of *Mrp2*, *Mrp3* and *Mrp4*. **D-F**, The hepatic mRNA levels of *GSTa1*, *GSTm1*, and *GSTm2*. Data are mean ±SEM, n = 5 for saline groups, n = 7 for APAP groups.

Supplementary Figure 5. Montelukast inhibit Cysltr1 expression in APAP-treated hepatocytes.
A, Primary hepatocytes were pretreated with montelukast (1, 5 and 10 μM) or vehicle (DMSO) 1 hour before APAP (2.5 mM) administration. Quantification of LDH released into the culture medium of primary hepatocyte after treatment with 2.5 mM of APAP for 24 hours. Data are mean ± SEM, n=3 for each group, $**p < 0.01$. **B**, Primary hepatocytes were pretreated with montelukast (5 μM) or vehicle (DMSO) 1 hour before APAP (2.5 mM) administration. Western blot analysis of Cysltr1. Experiments were repeated 3 times with similar results.

Supplementary Figure 6. Zafirlukast inhibit APAP-induced cell damage.
A-B, Primary hepatocytes were pretreated with zafirlukast (5 μM) or vehicle (DMSO) 1 hour before APAP (2.5 mM) administration. **A**, Quantification of LDH released into the culture medium of primary hepatocyte after treatment with 2.5 mM of APAP for 24 hours. Data are mean ±SEM, n = 3 for each group, $**p < 0.01$. **B,** Primary hepatocytes were incubated with 5mg/L JC-1 dye for 30 min at 37°C in the dark and washed twice with the dye buffer. Then the cells were quickly subjected to fluorescence microscope for captured red or green fluorescence. Experiments were repeated 3 times with similar results.

Supplementary Figure 7. Montelukast inhibit LTD_4-induced cell death.
A-C, Primary hepatocytes were pretreated with montelukast (5 μM) or vehicle (DMSO) 1 hour before LTD_4 (100 nM) administration. **A**, Representative morphological images of primary hepatocytes treated with LTD_4 for 24 hours. **B**, Quantification of LDH released into the culture medium of primary

hepatocyte after treatment with 2.5 mM of APAP for 24 hours. Data are mean ±SEM, n = 3 for each group. *$p < 0.05$. **C,** Primary hepatocytes were incubated with 5mg/L JC-1 dye for 30 min at 37°C in the dark and washed twice with the dye buffer. Then the cells were quickly subjected to fluorescence microscope for captured red or green fluorescence. Experiments were repeated 3 times with similar results.

Supplementary Table 1: List of specific primers used for real-time quantitative PCR

Genes	Forward sequence(5'-3')	Reverse sequence(5'-3')
18s	TTGACTCAACACGGGAAACC	AGACAAATCGCTCCACCAAC
Blt1	ATGGCTGCAAACACTACATCTC	GACCGTGCGTTTCTGCATC
Blt2	ATGTCTGTCTGCTACCGTCC	AGCTCCATACTACGAAGCCAT
Cysltr1	CCTCTCCGTGTGGTCTATTATGT	ATGCAAACGAACCTGGCTTTT
Cysltr2	TGTCACCAGTGTCAGGAGTG	ACTTTTGAGGACTCAGCTCCAA
Mcp1	GCTGGAGAGCTACAAGAGGATC	GTCAACTTCACATTCAAAGGTGC
Tnf-α	GGCGGTGCCTATGTCTCA	AGGGTCTGGGCCATAGAA
Il-6	TTCTTGGGACTGATGCTG	CTCATTTCCACGATTTCCC
Il-18	CACATGCGCCTTGTGATGAC	TGCAGCCTCGGGTATTCTG

Supplementary Figure 1

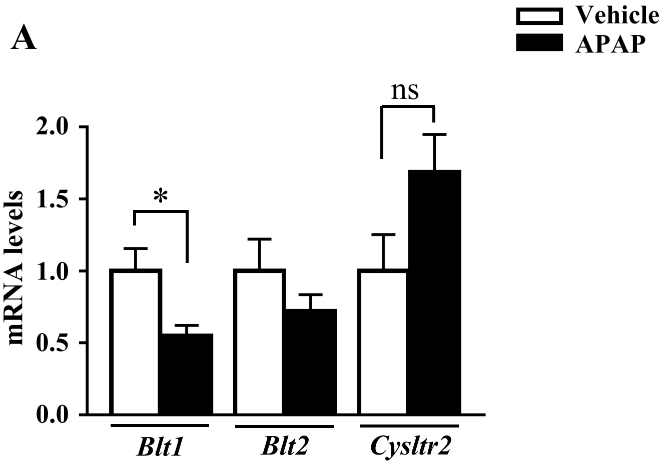

Supplementary Figure 2

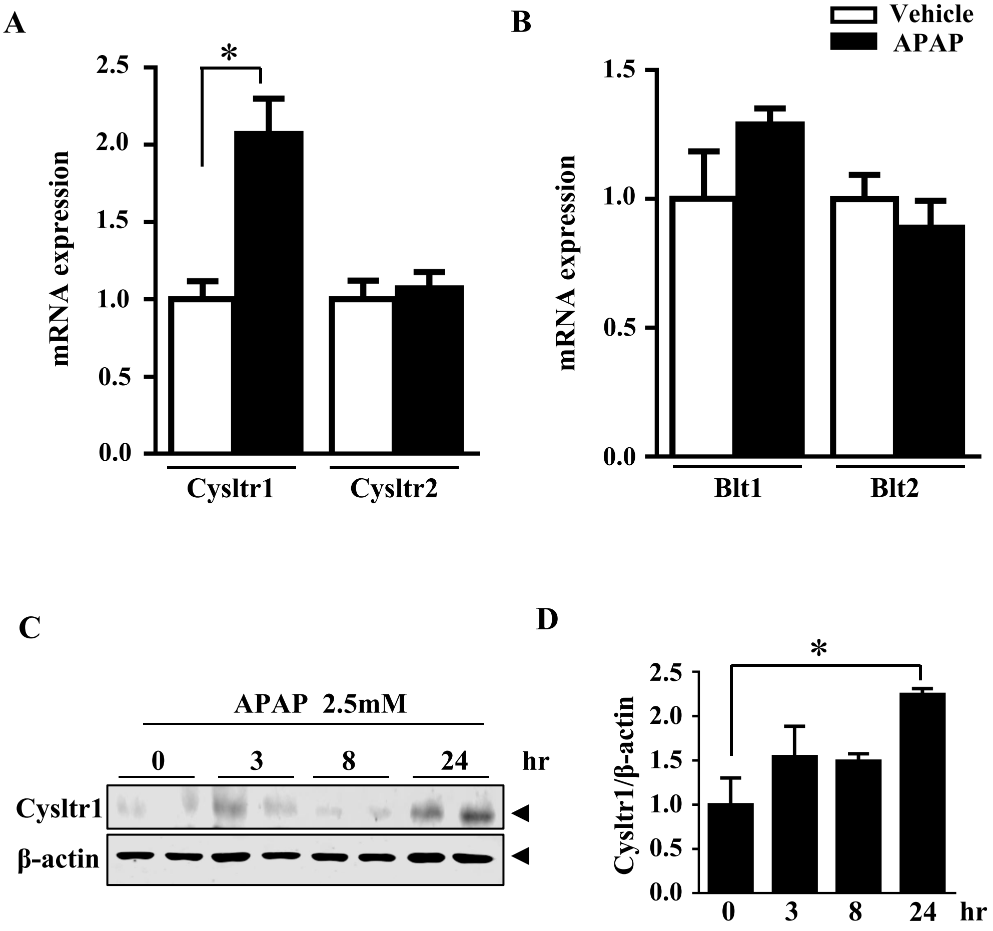

Supplementary Figure 3

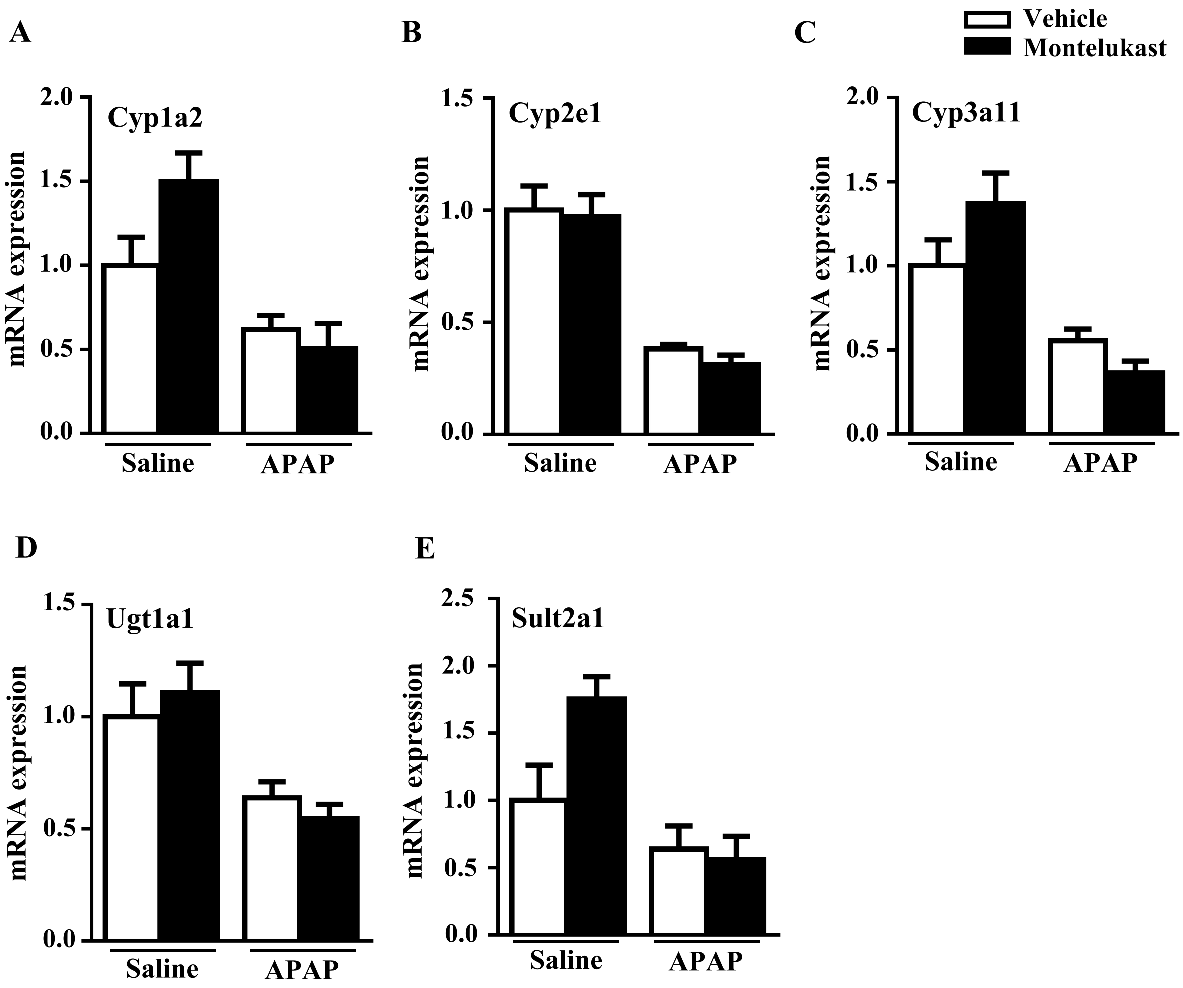

Supplementary Figure 4

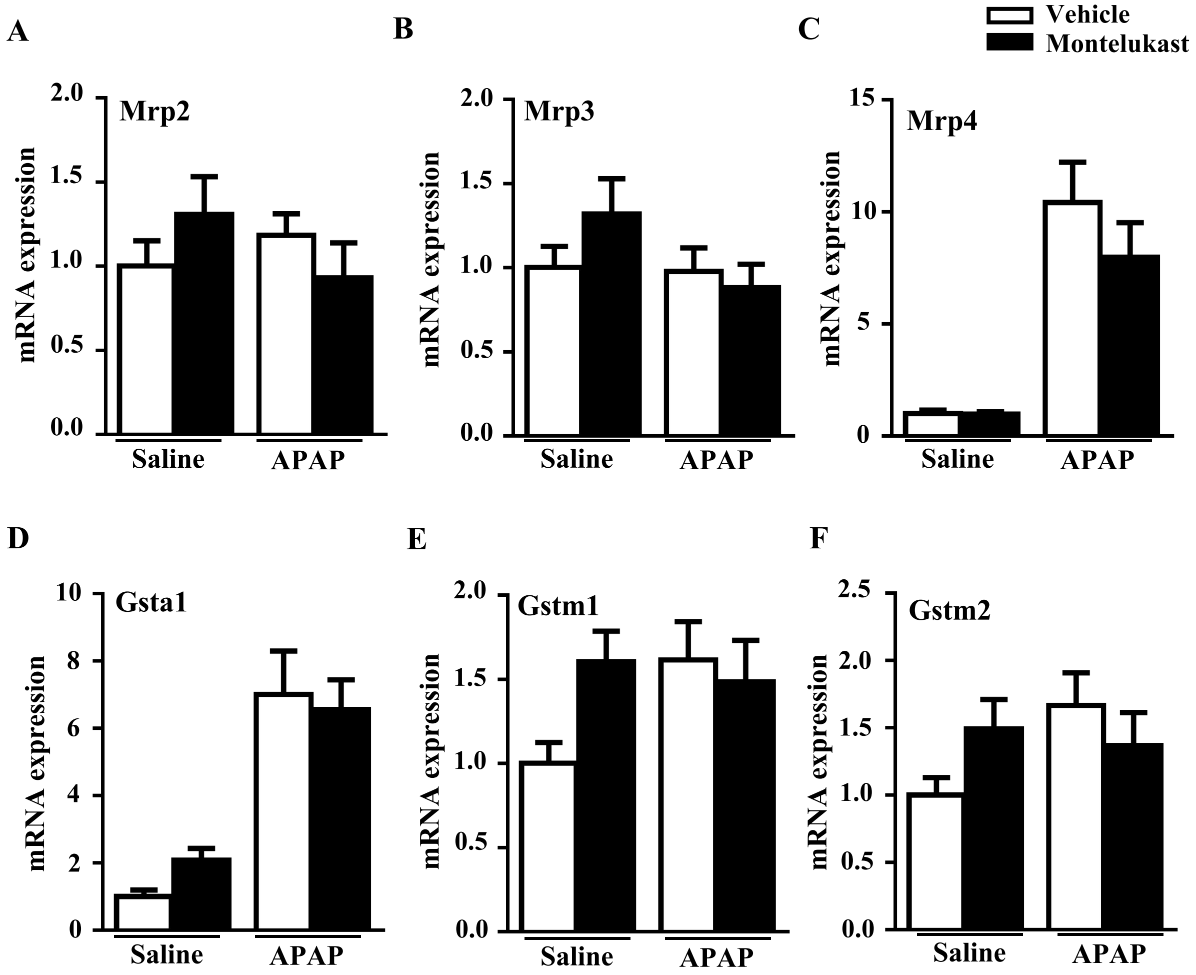

Supplementary Figure 5

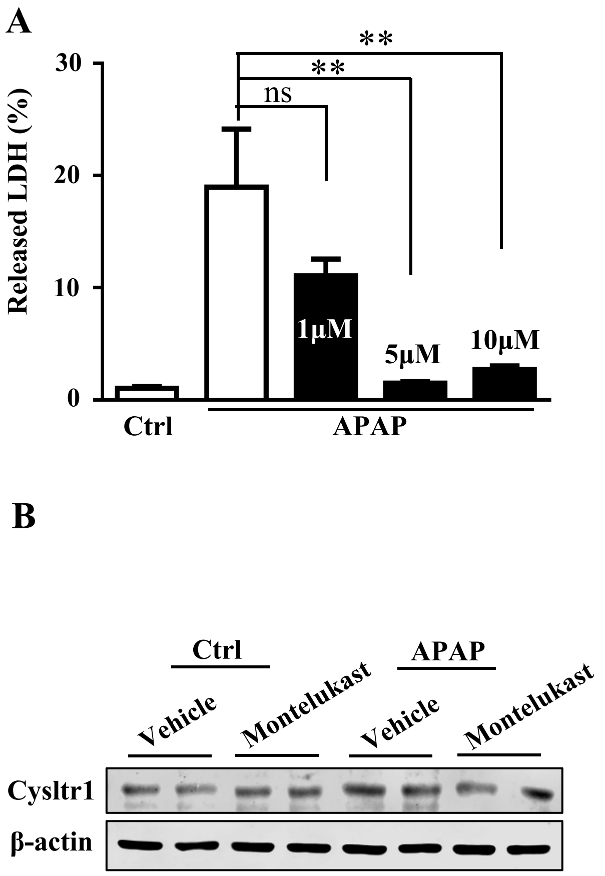

Supplementary Figure 6

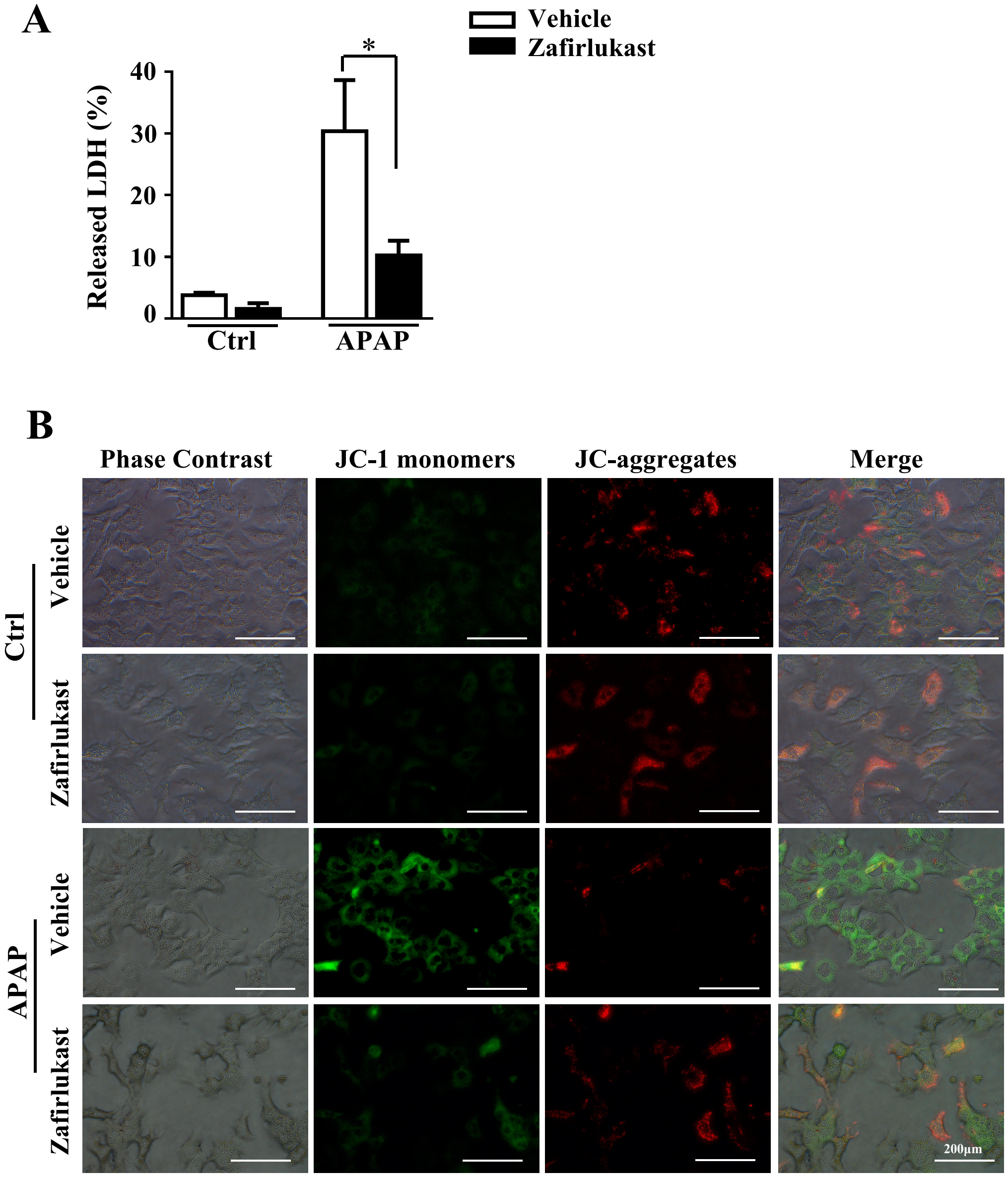

Supplementary Figure 7

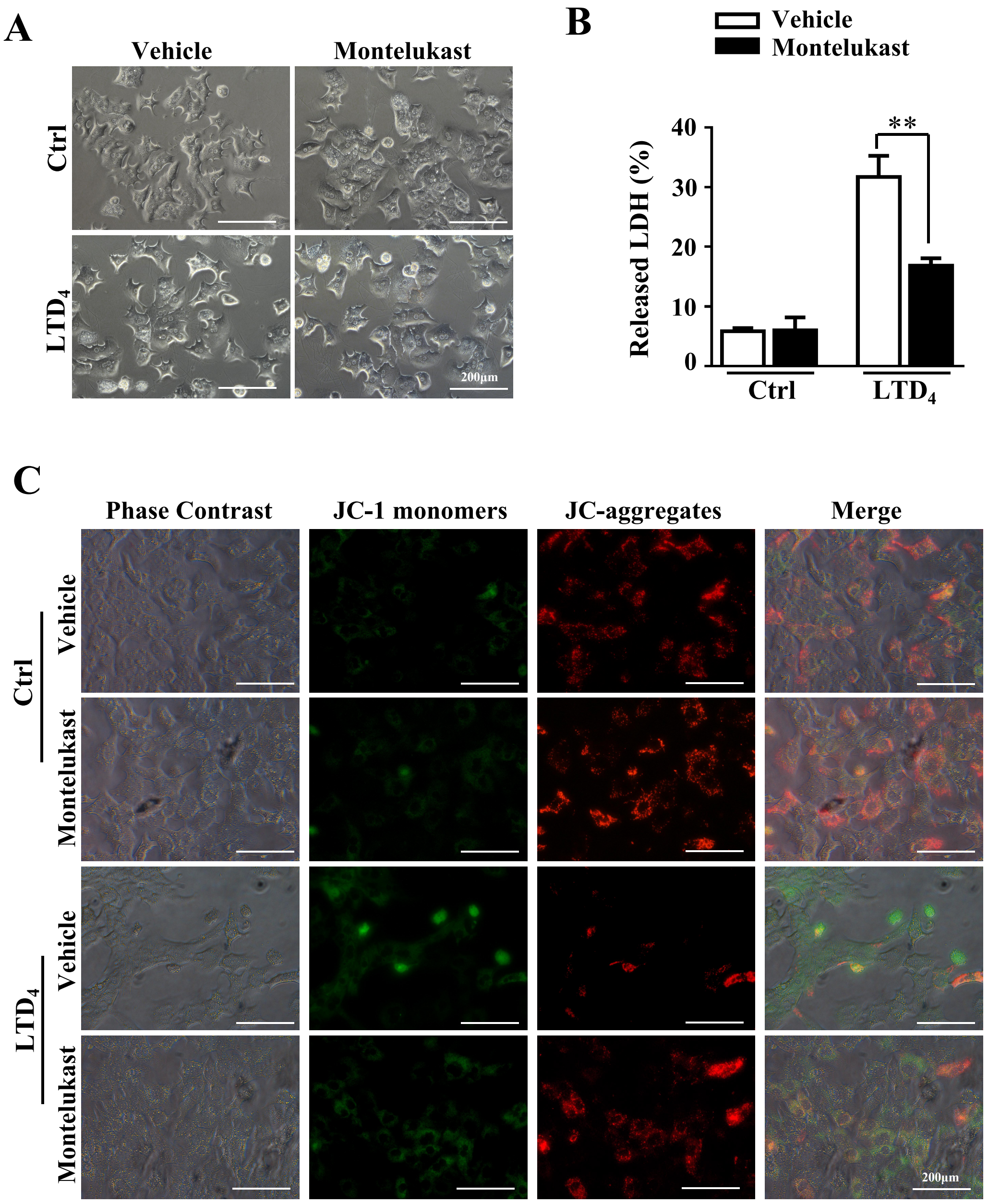

Supplement: Supplementary file 1 [file DataSheet_1.pdf]
